# Cephalometric Analysis of the Facial Skeletal Morphology of Female Patients Exhibiting Skeletal Class II Deformity with and without Temporomandibular Joint Osteoarthrosis

**DOI:** 10.1371/journal.pone.0139743

**Published:** 2015-10-16

**Authors:** Shuo Chen, Jie Lei, Kai-Yuan Fu, Xing Wang, Biao Yi

**Affiliations:** 1 Department of Oral and Maxillofacial Surgery, Peking University School and Hospital of Stomatology, Beijing, PR China; 2 Department of Oral & Maxillofacial Radiology, Peking University School and Hospital of Stomatology, Beijing, PR China; Medical University of South Carolina, UNITED STATES

## Abstract

**Purpose:**

This study evaluated the differences in the facial morphological characteristics of female patients exhibiting skeletal class II deformity with and without temporomandibular joint osteoarthrosis.

**Methods:**

Eighty-three female patients with skeletal class II deformity were included in this study; these patients were classified into three groups on the basis of the condylar features shown in cone-beam computed tomography scans: normal group, indeterminate for osteoarthrosis group, and osteoarthrosis group. The cephalometric differences among the three groups were evaluated through one-way ANOVA.

**Results:**

Of the 83 patients, 52.4% were diagnosed with osteoarthrosis, as indicated by the changes in the condylar osseous component. The cephalometric measurements that represented skeletal characteristics, including mandibular position relative to the cranial base, mandibular plane angle (MP-SN), posterior facial height (S-Go), and facial height ratio, were significantly different among the three groups (p < 0.05). The patients in the osteoarthrosis group yielded the smallest S-Go, the highest MP-SN, and the most retruded mandible.

**Conclusions:**

Temporomandibular joint osteoarthrosis is commonly observed in female patients with skeletal class II deformity. The morphological characteristics of the facial skeleton in patients with bilateral condylar osteoarthrosis may be altered.

## Introduction

Temporomandibular joint osteoarthrosis (TMJOA), a subtype of temporomandibular disorders (TMD), has been considered as a degenerative change in articular cartilage and subchondral bone [1]. The clinical signs and symptoms of TMJOA lack specificity and evidence; as such, TMJOA may be diagnosed through imaging examination [2]. Ahmad et al. [3] developed a set of image analysis criteria as a part of the research diagnostic criteria for TMDs (RDC/TMD). The evaluation results of the TMJ osseous component are recorded on a scoring form that indicates remodeling or degenerative changes in bone. After observations are completed, a diagnosis is performed to categorize the joint as normal, indeterminate, or affected with osteoarthrosis.

The relationship between TMD and dentofacial deformity has been investigated [4–9]. Patients with class II deformity are prone to internal derangements [4] and osteoarthrosis [9]. Deformities are more severe in disk displacement without reduction than those in disk displacement with reduction [5]. However, the differences in the facial morphological characteristics of patients exhibiting skeletal class II deformity with and without TMJOA have not been explored. Female patients are more vulnerable to TMD than males [10–13]. Therefore, our study aimed to evaluate condylar joint conditions through cone-beam computed tomography (CBCT); this study also aimed to analyze the differences in the facial morphological characteristics of female patients exhibiting skeletal class II deformity with and without TMJOA.

## Materials and Methods

### Subjects

Eighty-three patients (mean age, 24.1 ± 3.4 years; range, 18.0–30.0 years), who underwent orthodontic treatment and subsequent orthognathic surgery in our hospital from January 1, 2011 to December 31, 2013 were included in this study. All of the patients seeking treatment were informed of the possibility that their medical records might be used for teaching or research purposes; hence, verbal consent was obtained. In this retrospective study, patients’ data and radiographs were selected from the database of our hospital and anonymized for analysis. The study protocol was approved by the Institutional Review Board of Peking University School and Hospital of Stomatology (Approval no. PKUSSIRB-2012012). All of the patients were diagnosed with skeletal class II and angle class II, division 1 malocclusion without anterior open bite. Patients who manifested severe facial asymmetry, deformity secondary to trauma, ankylosis, or systemic disease were excluded from the study.

### Image data acquisition

**Fig 1:**
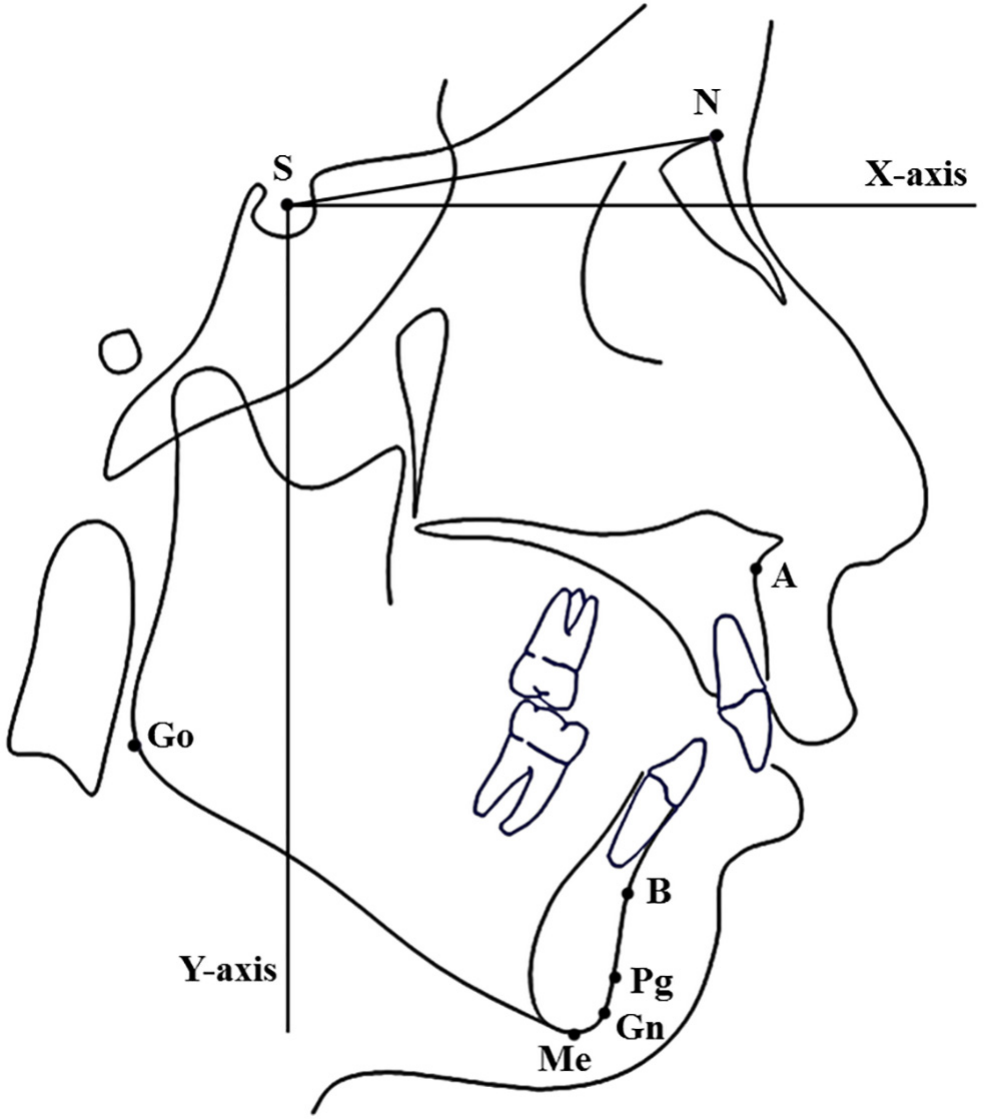
Coordinate system and hard tissue landmarks used in cephalometric analysis.

The CBCT scans of TMJs and standardized lateral cephalograms were obtained during the initial appointment. The cephalograms were digitized and analyzed (Dolphin Imaging and Management Solutions, Chatsworth, CA, USA) by an independent examiner who was blinded to the patients’ TMJ diagnostic results. As a basis of measurements, an X–Y cranial base coordinate system was constructed on the radiographs (Fig 1). An X-axis was drawn 7° to the sella–nasion line (SN); the Y-axis was illustrated along the sella perpendicular to the X-axis [14]. The definitions of cephalometric landmarks and some measurements are presented in Table 1 and Fig 1. The measurements were repeated thrice, and the mean value was statistically analyzed.

**Table 1:** Cephalometric landmarks and some measurement definitions.

Landmark	Definition
Sella (S)	The center of sella turcica
Nasion (N)	The most anterior point of the frontonasal suture
Point-A (A)	The innermost point on the contour of the maxilla between the anterior nasal spine and the incisor
Point-B (B)	The innermost point on the contour of the mandible between the incisor and the bony chin
Gonion (Go)	The point on the curvature of the angle of the mandible located by bisecting the angle formed by the lines tangent to the posterior ramus and the inferior border of the mandible
Pogonion (Pg)	The most anterior point on the chin
Menton (Me)	The most inferior point on the mandibular symphysis in the midline
Gnathion (Gn)	The lowest, most anterior midline point on the symphysis of the mandible (midway between the menton and the pogonion)
A to Y-axis distance	The vertical distance from point A to Y-axis
Pg to Y-axis distance	The vertical distance from point Pg to Y-axis
Mandibular plane (MP)	Extends from the gonion to the gnathion
Mandibular plane angle (MP-SN)	The angle between mandibular plane and SN
Anterior facial height (N-Me)	The distance between the point nasion and the point menton perpendicular to the X-axis
Posterior facial height (S-Go)	The vertical distance from the point gonion to the X-axis
Facial height ratio (S-Go/N-Me)	The ratio between S-Go and N-Me

The CBCT machine (DCT Pro; Vatech, Seoul, Korea) was used to evaluate condylar osseous conditions. All of the patients sat in an upright position with the teeth in the centric occlusion. The patients’ Frankfort horizontal plane was parallel to the floor. The scanning settings of the CBCT machine were as follows: 16 cm × 10 cm field of view; 90 kVp tube voltage; 7.0 mA tube current; and 24 s scan time. The CBCT data were reconstructed using the 3D image dental software Ez3D2009 Simple Viewer Ver. 1.2.1.0 (E-WOO Technology Co., Seoul, Korea) in accordance with a previously described method [15].

The condylar images were categorized into three groups on the basis of the classification of the osseous diagnosis for TMJ, as proposed by Ahmad [3] and Ma [16].

1. Normal

**Fig 2:**
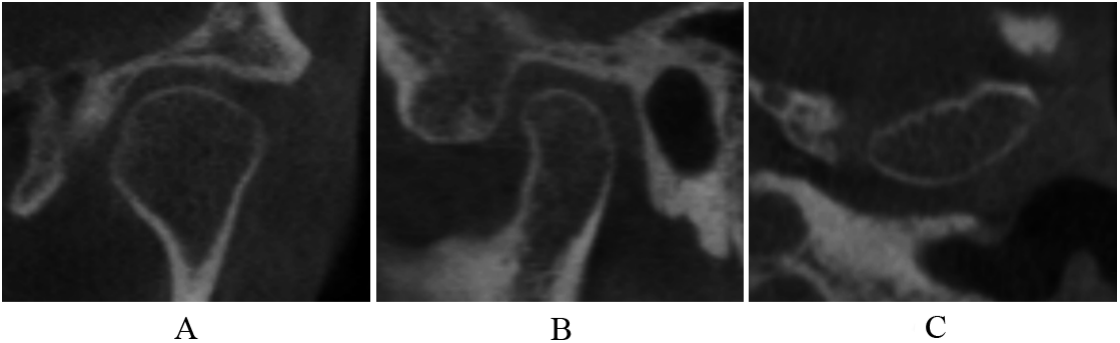
Normal condyle in coronal (A), sagittal (B), and axial (C) images.

Normal relative size of the condylar head; no subcortical sclerosis or articular surface flattening; and no deformation caused by subcortical cyst, surface erosion, osteophyte, or generalized sclerosis (Fig 2).

2. Indeterminate for osteoarthrosis

**Fig 3:**
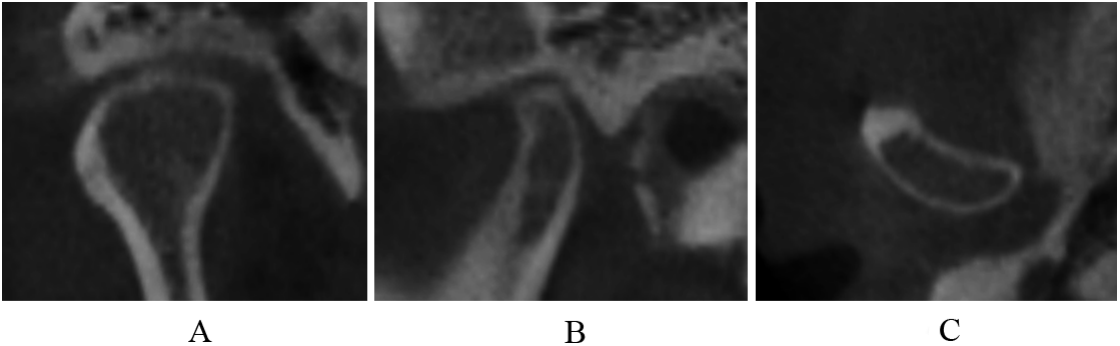
Subcortical sclerosis in coronal (A), sagittal (B), and axial (C) images.

**Fig 4:**
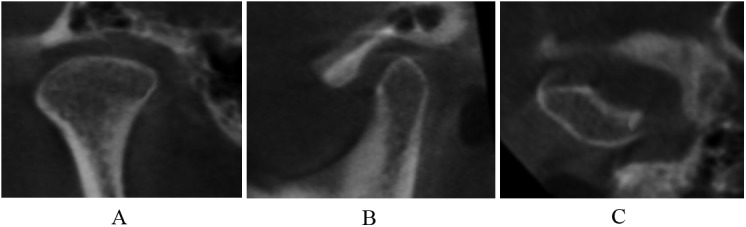
Articular surface flattening in coronal (A), sagittal (B), and axial (C) images.

**Fig 5:**
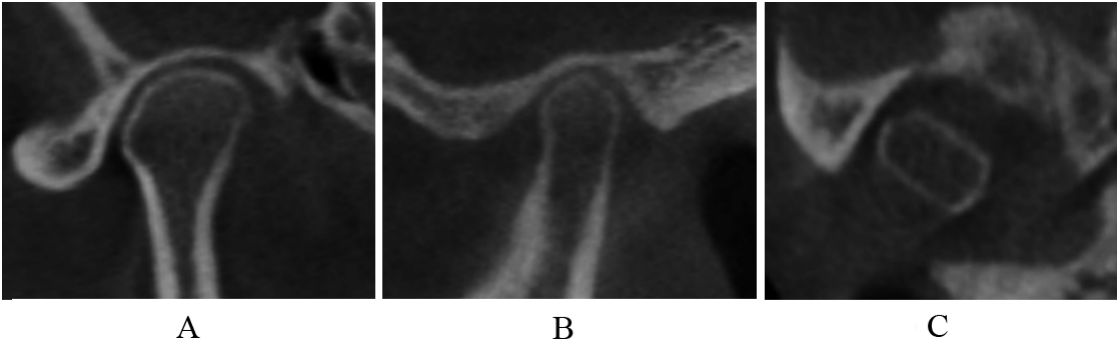
Condylar hypoplasia in coronal (A), sagittal (B), and axial (C) images.

Normal relative size of the condylar head; subcortical sclerosis with or without articular surface flattening (Fig 3); articular surface flattening with or without subcortical sclerosis (Fig 4); no deformation caused by subcortical cyst, surface erosion, osteophyte, or generalized sclerosis; and condylar hypoplasia with normal condylar morphology but decreased size in all dimensions (Fig 5).

3. Osteoarthrosis

**Fig 6:**
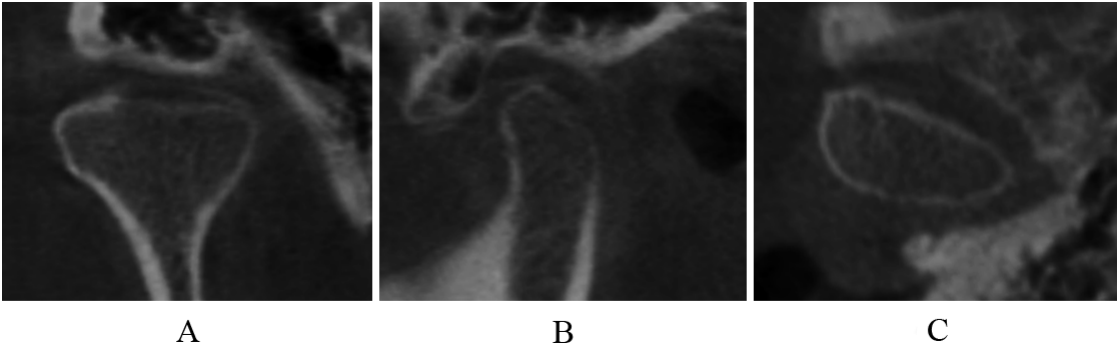
Surface erosion in coronal (A), sagittal (B), and axial (C) images.

**Fig 7:**
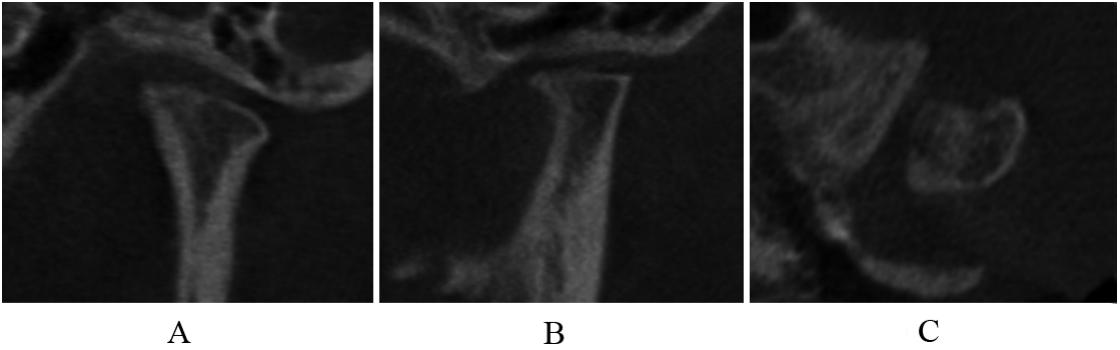
Osteophyte and short condyles in coronal (A), sagittal (B), and axial (C) images.

**Fig 8:**
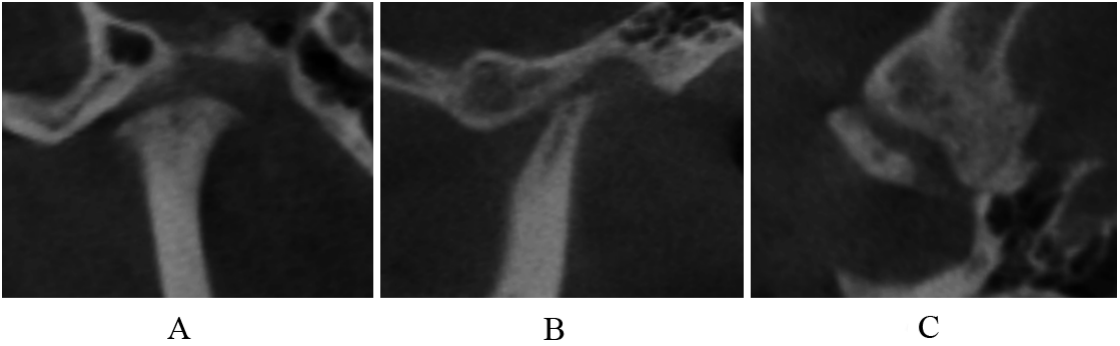
Generalized sclerosis and short condyles in coronal (A), sagittal (B), and axial (C) images.

**Fig 9:**
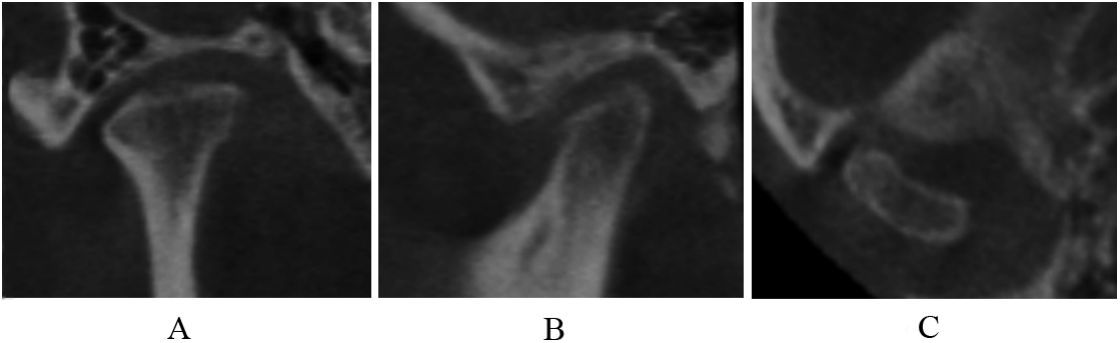
Short condyles in coronal (A), sagittal (B), and axial (C) images.

Deformation caused by subcortical cyst, surface erosion (Fig 6), osteophyte (Fig 7), or generalized sclerosis (Fig 8); and short condyles with decreased head height but continuous articular cortex (Fig 9).

Changes in osseous tissues were documented by two independent assessors. In case of disagreement, a final diagnosis was obtained by consensus. Changes in bones were observed in at least two consecutive slices.

### Statistical analysis

Data were statistically analyzed using SPSS (version 13.0 for Windows). Fifteen cases were selected randomly, and the same investigator repeated the measurements at least after two weeks to assess the reliability of the method. Paired *t* test was conducted to assess systematic error; Dahlberg formula [17] was used to calculate random error.

The cephalometric differences among the three groups were evaluated through one-way ANOVA. The LSD multiple comparison test was performed (p = 0.05) when equal variances were assumed. Dunnett’s T3 multiple comparison test was conducted when equal variances were not assumed.

## Results

The random errors of linear measurements ranged from 0.61 mm to 0.79 mm; the random errors of angular measurements ranged from 0.37° to 0.64°. The paired *t* test showed no significant differences at p = 0.05.

Overall, 54.2% (*n* = 45) of the patients were diagnosed with osteoarthrosis, as indicated by the changes in the condylar osseous component. Six patients suffered from unilateral osteoarthrosis; of these six patients, three showed normal condyle and three were indeterminate for osteoarthrosis in the contralateral side. Two patients were indeterminate for osteoarthrosis unilateral and normal in the contralateral side. Thirty-nine patients exhibited bilateral osteoarthrosis, nineteen patients were indeterminate for bilateral osteoarthrosis, and seventeen patients displayed normal bilateral condyles (Table 2). Only the subjects with normal bilateral condyles (group 1), indeterminate for bilateral osteoarthrosis (group 2), and bilateral osteoarthrosis (group 3) were included in the study to evaluate the relationship between condylar image features and facial morphological characteristics. The general findings of different groups were showed in Table 3.

**Table 2:** Distribution of patients on the basis of condylar osseous features.

Group*	Unilateral	Bilateral	Total
Normal	0	17	17
Indeterminate	2	19	21
Osteoarthrosis	6	39	45
Total	8	75	83

* When bilateral condylar imaging diagnosis was different, the serious side was determined as the diagnosis of the patient.

**Table 3:** Clinical features of the normal group, the indeterminate group, and the osteoarthrosis group.

	Normal (n = 17)	Indeterminate(n = 19)	Osteoarthrosis (n = 39)
Age(year), mean ± SD	25.1±2.3	23.7±3.2	23.8±3.9
Mouth opening(mm), mean ± SD	42.0±2.7	42.4±3.0	41.0±3.0
Joint noise	5	7	11
Tender of joint	0	0	1

Table 4 shows the distribution of osseous diagnoses of all condyles. Short condyles (18.6%) were the most common signs of osteoarthrosis, followed by osteophyte (15.3%), surface erosion (14.2%), and generalized sclerosis (7.1%). Subcortical cyst was not detected.

**Table 4:** Prevalence of the condylar osseous features revealed by CBCT.

Features ^#^	N	%
Normal	39	21.3
Condylar hypoplasia	15	8.2
Articular surface flattening	17	9.3
Subcortical sclerosis	11	6.0
Subcortical cyst	0	0
Erosion	26	14.2
Osteophyte	28	15.3
Generalized sclerosis	13	7.1
Short condyle	34	18.6
Total	183	100

^#^ When the condyle presented different osseous features, each feature is recorded.

The differences in cephalometric measurements that represented skeletal characteristics, including SNB, ANB, mandibular plane angle (MP-SN), Pg to Y-axis distance, posterior facial height (S-Go), and facial height ratio (S-Go/N-Me), were statistically significant among the three groups (p < 0.05; Table 5).

**Table 5:** Comparison of the cephalometric variables of the normal group, the indeterminate group, and the osteoarthrosis group.

	Normal (*n* = 17)	Indeterminate (*n* = 19)	Osteoarthrosis (*n* = 39)
Angular (°)			
SNA	82.40±3.63^a^	82.80±2.33 ^a^	81.94±2.87 ^a^
SNB	73.94±3.52 ^a^	73.14±2.74 ^a^ ^,^ ^b^	71.89±2.56^b^
ANB	8.45±1.50 ^a^	9.66±2.71 ^a^ ^,^ ^b^	10.05±1.96 ^b^
MP-SN	39.11±6.73^a^	42.84±4.13 ^a^	47.97±6.53^b^
Linear (mm)			
A to Y-axis	61.69±3.69 ^a^	60.84±4.11 ^a^	59.56±4.71 ^a^
Pg to Y-axis	48.60±6.46 ^a^	45. 12±4.16 ^a^	41.38±5.78 ^b^
S-Go	78.01±6.96 ^a^	74.72±6.11 ^a^	68.33±4.63^b^
N-Me	122.25±8.45 ^a^	123.74±5.91 ^a^	119.81±5.45 ^a^
S-Go/N-Me(%)	63.89±4.76 ^a^	60.37±3.88 ^b^	57.13±4.48^c^

^a,b,c^ The same superscripts indicate no statistically significant differences among the indicated groups (p > 0.05). LSD multiple comparison test was used when equal variances were assumed; Dunnett’s T3 multiple comparison test was used when equal variances were not assumed.

## Discussion

In this study, the radiographic features of condylar bony conditions and facial skeletal morphological characteristics of the female patients who manifested skeletal class II deformity were evaluated. Results showed that more than half of the patients suffered from osteoarthrosis. The patients with osteoarthrosis exhibited the smallest S-Go, highest MP, and the most retruded mandible among the three groups.

CT provides optimal imaging of the osseous components of TMJ. However, the high cost and relatively high radiation dose have limited the widespread use of CT to detect condylar changes. Recently developed CBCT has been extensively applied in the oral and maxillofacial region because of its lower radiation dose and lower cost [18]. The CBCT images are superior to traditional TMJ radiographs [19,20] and similar to spiral CT in detecting bony changes [21]. Therefore, CBCT can be an optimal method to evaluate condylar bony components.

With the widespread use of CBCT, a high prevalence of degenerative bone alteration has been detected in patients who underwent TMD treatment [11–13], orthodontic treatment [22], or orthognathic surgery [9]; this condition is more frequent in females than in males. Krisjane [9] compared the prevalence of TMJOA in patients with different dentofacial deformities. An image feature that corresponded to a diagnosis of osteoarthrosis was observed in 43% of class II joints, which showed a significantly higher percentage than class I and class III groups (3% and 20% respectively).

Our study focused on the condylar bony conditions of female patients with skeletal class II deformity. The occurrence of osteoarthrosis in the group was 54.2%. A short condylar process was the most prevalent feature in the osteoarthrosis group. This type is characterized with decreased head height but continuous articular cortex [16]. Short condyles, osteophytes, and generalized sclerosis may indicate late and relatively stable stages; by contrast, erosive lesions may indicate early or active stages of degenerative changes [23].

The cephalometric variables showed that the mandibular position relative to the cranial base was significantly different; by contrast, the maxillary position (SNA and point A to Y-axis distance) was similar. In addition, the difference in S-Go/N-Me resulted from smaller S-Go because the differences in the measurements of the anterior facial height (N-Me) did not show any significance among the three groups. This study showed that the facial morphology was more different between the normal group and the osteoarthrosis group than between the normal group and the indeterminate group. The patients with osteoarthrosis yielded the smallest S-Go, highest MP-SN, and the most retruded mandible, exhibiting a clockwise rotation tendency. This facial morphology in skeletal class II deformity with osteoarthrosis may have resulted from a reduced condylar growth potential. The pathological process of TMJOA is characterized by the collapse and degeneration of cartilage and the underlying bone [1]. Condylar cartilage plays a vital role in the growth of condyle and mandible [24]. Degenerative joint disease in adolescents likely diminishes the growth potential of the mandible; as a result, short ramus height and mandibular deficiency can be observed. The reduced condylar growth may enhance the development of N-Me from nasomaxillary complex growth and dentoalveolar development [25]. Thus, an imbalance in S-Go/N-Me, increased MP-SN, and clockwise rotation of the mandible are observed [26].

Although our study suggested that osteoarthrosis of the TMJ likely affects skeletal morphology, the cause-and-effect relationship remains unclear. The hyperdivergent facial morphology may play a role in the development of TMJOA. The stability of the TMJ disc depends on the integrity of the joint ligaments and the forces applied over the TMJ. Hyperdivergent facial morphology is prone to disc displacement because of the poor reciprocal fitting of the articular surfaces, that is, less contact area is observed between condylar head and glenoid fossa compared with hypodivergent facial morphology [27]. The disc-dislocation stretching forces from the lateral pterygoid muscle may increase in patients with a hyperdivergent facial pattern [27]. This deduction is supported by findings in a previous study, which showed that the internal derangement of the TMJ is much more prevalent in subjects with more posteriorly rotated mandibular ramus, smaller mandible, and steeper mandibular planes [6]. Internal derangement, especially disk displacement without reduction, may progress to osteoarthrosis [28]. Therefore, hyperdivergent facial morphology may be a risk factor of the development of TMJOA.

The TMJ is considered as a constant remodeling stage. Functional remodeling such as articular surface flattening and subcortical sclerosis, is characterized by the adaptation of the articular structures of the TMJ in response to mechanical stress. Degenerative changes, such as surface erosion, osteophyte, and generalized sclerosis, may occur when the balance between the host adaptive capacity and the mechanical loading on the condyle is lost. Condyles with degenerative changes may cause an unfavorable response to orthodontic treatment or orthognathic surgery. Thus, orthodontists or surgeons should focus on condylar bony conditions in female patients with skeletal class II deformity, especially those who presented hyperdivergent facial morphology.

## Conclusions

In this study, the radiographic features of condyles were evaluated through CBCT. More than half of the patients were diagnosed with osteoarthrosis in the condylar osseous component. Patients with osteoarthrosis yielded the smallest S-Go, the highest MP-SN, and the most retruded mandible. Clinicians should be aware that some cephalometric variables may indicate the potential osteoarthrosis of condyle.

## Supporting Information

S1 Table Cephalometric measurements of the subjects in the normal group, the indeterminate group, and the osteoarthrosis group. (DOCX)

S2 Table Repeated cephalometric measurements at least 2 weeks apart to assess reliability of the method. (DOCX)
