# Supplementary material for: Cephalometric Analysis of the Facial Skeletal Morphology of Female Patients Exhibiting Skeletal Class II Deformity with and without Temporomandibular Joint Osteoarthrosis
Source: PLoS One. 2015 Oct 16;10(10):e0139743. doi: 10.1371/journal.pone.0139743 (PMC4608765; doi:10.1371/journal.pone.0139743)
Supplement: S1 Table — (DOCX) [file pone.0139743.s001.docx]

**Supporting Information**

**(S1)**

**Table 1.cephalometric measurements of subjects with Normal group, Indeterminate group, and Osteoarthrosis group**

| **No.** | **SNA** | **SNB** | **ANB** | **A** | **Pog** | **MP -SN** | **SGo** | **NaMe** | **PA** |
| --- | --- | --- | --- | --- | --- | --- | --- | --- | --- |
| 1 | 87.3 | 78.1 | 9.2 | 63.7 | 50.1 | 35.6 | 86.9 | 127.4 | 68.2 |
| 2 | 85.2 | 75.6 | 9.6 | 62.4 | 49.5 | 36.8 | 71.5 | 109.8 | 65.1 |
| 3 | 80.3 | 72.0 | 8.3 | 59.7 | 41.1 | 53.1 | 67.8 | 127.6 | 53.1 |
| 4 | 85.2 | 73.7 | 11.5 | 65.2 | 51.9 | 47.8 | 84.5 | 137.9 | 61.3 |
| 5 | 84.7 | 74.8 | 9.9 | 59.8 | 42.9 | 42.8 | 74.4 | 119.4 | 62.3 |
| 6 | 82.8 | 75.0 | 7.9 | 67.4 | 58.2 | 27.1 | 85.3 | 117.9 | 72.4 |
| 7 | 77.1 | 71.1 | 5.9 | 57.1 | 43.7 | 41.0 | 74.1 | 121.4 | 61.0 |
| 8 | 77.7 | 69.7 | 8.0 | 58.8 | 43.6 | 42.9 | 68.7 | 116.2 | 59.2 |
| 9 | 81.5 | 73.1 | 8.4 | 54.4 | 40.2 | 41.1 | 72.0 | 113.2 | 63.6 |
| 10 | 81.3 | 73.0 | 8.3 | 58.7 | 51.8 | 33.5 | 86.8 | 127.6 | 68.0 |
| 11 | 84.6 | 75.0 | 9.6 | 64.0 | 47.4 | 45.0 | 75.6 | 125.2 | 60.4 |
| 12 | 85.3 | 77.1 | 8.2 | 67.4 | 56.1 | 36.0 | 78.8 | 122.9 | 64.1 |
| 13 | 76.7 | 68.8 | 7.9 | 58.4 | 46.6 | 37.2 | 67.9 | 111.4 | 61.0 |
| 14 | 83.2 | 74.9 | 8.3 | 65.0 | 57.5 | 28.1 | 81.5 | 114.9 | 71.0 |
| 15 | 84.3 | 74.8 | 9.5 | 64.0 | 46.4 | 42.8 | 84.7 | 135.3 | 62.6 |
| 16 | 76.4 | 68.0 | 8.3 | 59.9 | 39.7 | 41.0 | 85.5 | 133.6 | 64.0 |
| 17 | 87.2 | 82.3 | 4.9 | 62.9 | 59.5 | 33.0 | 80.2 | 116.6 | 68.8 |
| 18 | 84.5 | 74.3 | 10.2 | 56.4 | 40.3 | 46.1 | 69.3 | 117.9 | 58.8 |
| 19 | 83.6 | 72.6 | 11.0 | 61.7 | 39.4 | 48.5 | 73.7 | 128.1 | 57.5 |
| 20 | 87.9 | 76.4 | 11.5 | 65.9 | 48.2 | 41.7 | 76.8 | 124.8 | 61.5 |
| 21 | 78.8 | 71.6 | 7.2 | 55.8 | 40.7 | 47.8 | 69.1 | 121.4 | 56.9 |
| 22 | 84.3 | 74.8 | 9.5 | 61.4 | 45.8 | 42.8 | 84.7 | 135.1 | 62.7 |
| 23 | 82.1 | 68.0 | 14.1 | 65.4 | 47.4 | 41.0 | 85.5 | 133.3 | 64.1 |
| 24 | 82.0 | 71.5 | 10.5 | 53.5 | 47.9 | 38.8 | 68.3 | 118.6 | 57.6 |
| 25 | 81.1 | 78.5 | 2.6 | 61.4 | 51.8 | 40.4 | 82.7 | 123.9 | 66.7 |
| 26 | 80.4 | 70.1 | 10.3 | 61.2 | 43.8 | 41.5 | 68.3 | 118.7 | 57.5 |
| 27 | 81.9 | 70.3 | 11.6 | 61.8 | 42.8 | 38.8 | 78.3 | 120.6 | 64.9 |
| 28 | 83.9 | 73.9 | 10.0 | 55.4 | 47.8 | 39.3 | 73.4 | 126.9 | 57.8 |
| 29 | 82.0 | 71.4 | 10.6 | 63.4 | 50.8 | 38.5 | 68.3 | 119.7 | 57.1 |
| 30 | 87.3 | 77.1 | 10.2 | 57.4 | 39.8 | 36.4 | 76.8 | 118.5 | 64.8 |
| 31 | 81.0 | 71.2 | 9.8 | 66.4 | 41.8 | 39.1 | 68.3 | 129.7 | 52.7 |
| 32 | 82.5 | 71.5 | 11.0 | 61.1 | 47.8 | 48.8 | 76.8 | 119.7 | 64.2 |
| 33 | 82.8 | 75.1 | 7.7 | 68.4 | 47.8 | 47.0 | 81.6 | 133.1 | 61.3 |
| 34 | 84.9 | 73.9 | 11.0 | 56.4 | 47.8 | 49.3 | 73.4 | 126.2 | 58.2 |
| 35 | 79.8 | 76.1 | 3.7 | 61.4 | 37.8 | 46.3 | 66.0 | 115.2 | 57.3 |
| 36 | 82.3 | 71.3 | 11.0 | 61.5 | 47.8 | 41.8 | 78.3 | 119.7 | 65.4 |
| 37 | 87.3 | 77.3 | 10.0 | 67.1 | 51.3 | 36.4 | 76.8 | 118.5 | 64.8 |
| 38 | 82.1 | 74.3 | 7.7 | 60.5 | 46.9 | 36.1 | 74.3 | 115.3 | 64.4 |
| 39 | 81.2 | 70.7 | 10.5 | 61.4 | 42.3 | 48.0 | 66.1 | 118.9 | 55.6 |
| 40 | 79.3 | 73.8 | 5.5 | 51.7 | 40.6 | 44.2 | 65.8 | 110.7 | 59.4 |
| 41 | 81.5 | 72.1 | 9.4 | 59.0 | 42.0 | 45.4 | 70.1 | 120.9 | 58.0 |
| 42 | 83.5 | 71.5 | 12.0 | 55.4 | 37.2 | 52.8 | 63.9 | 117.8 | 54.3 |
| 43 | 83.6 | 75.0 | 8.6 | 65.1 | 51.4 | 45.3 | 62.6 | 115.7 | 54.1 |
| 44 | 79.2 | 68.3 | 10.8 | 54.6 | 38.3 | 47.6 | 69.7 | 121.3 | 57.5 |
| 45 | 82.4 | 72.1 | 10.3 | 64.6 | 37.9 | 55.0 | 71.6 | 131.3 | 54.6 |
| 46 | 81.7 | 74.1 | 7.7 | 59.6 | 42.0 | 49.1 | 72.4 | 128.8 | 56.2 |
| 47 | 80.4 | 70.1 | 10.4 | 61.1 | 42.1 | 45.5 | 68.3 | 118.4 | 57.7 |
| 48 | 85.4 | 71.7 | 13.7 | 57.5 | 32.3 | 55.0 | 65.1 | 117.8 | 55.3 |
| 49 | 83.2 | 73.4 | 9.8 | 61.3 | 42.9 | 50.2 | 59.6 | 113.0 | 52.8 |
| 50 | 74.3 | 67.2 | 7.1 | 51.1 | 33.3 | 51.0 | 61.2 | 118.9 | 51.5 |
| 51 | 83.2 | 73.9 | 9.3 | 62.4 | 44.7 | 51.2 | 61.6 | 118.2 | 52.1 |
| 52 | 79.8 | 69.1 | 10.7 | 54.7 | 35.1 | 54.3 | 66.0 | 124.9 | 52.8 |
| 53 | 83.6 | 73.5 | 10.1 | 62.3 | 45.7 | 34.8 | 75.6 | 114.0 | 66.3 |
| 54 | 86.0 | 72.0 | 14.0 | 67.8 | 44.0 | 47.3 | 71.3 | 124.9 | 57.1 |
| 55 | 80.1 | 69.3 | 10.8 | 56.1 | 34.0 | 52.2 | 70.5 | 124.7 | 56.5 |
| 56 | 85.1 | 72.4 | 12.8 | 58.9 | 38.7 | 44.2 | 68.6 | 111.8 | 61.4 |
| 57 | 81.5 | 68.5 | 13.0 | 56.9 | 40.3 | 53.4 | 62.7 | 123.9 | 50.6 |
| 58 | 80.1 | 71.8 | 8.3 | 61.0 | 46.0 | 45.5 | 70.0 | 121.0 | 57.9 |
| 59 | 82.3 | 72.8 | 9.5 | 55.2 | 37.7 | 50.2 | 66.2 | 117.7 | 56.2 |
| 60 | 80.3 | 71.0 | 9.3 | 63.9 | 48.3 | 44.2 | 64.0 | 113.6 | 56.3 |
| 61 | 84.3 | 73.1 | 11.1 | 55.8 | 40.8 | 46.1 | 66.1 | 112.9 | 58.5 |
| 62 | 83.3 | 72.8 | 10.5 | 60.4 | 41.2 | 55.6 | 63.0 | 124.0 | 50.8 |
| 63 | 77.7 | 72.3 | 5.5 | 53.6 | 41.8 | 40.5 | 74.9 | 115.0 | 65.1 |
| 64 | 78.4 | 67.6 | 10.8 | 55.7 | 39.8 | 48.3 | 68.4 | 121.4 | 56.4 |
| 65 | 77.6 | 69.0 | 8.6 | 56.7 | 34.6 | 61.3 | 68.2 | 130.9 | 52.1 |
| 66 | 86.3 | 76.0 | 10.2 | 67.7 | 53.2 | 38.8 | 72.5 | 115.6 | 62.8 |
| 67 | 83.9 | 73.7 | 10.2 | 64.5 | 43.5 | 49.3 | 73.4 | 126.9 | 57.8 |
| 68 | 85.5 | 74.1 | 11.4 | 68.0 | 48.6 | 40.7 | 76.4 | 122.4 | 62.4 |
| 69 | 82.3 | 71.1 | 11.1 | 54.8 | 32.8 | 48.3 | 66.1 | 112.9 | 58.5 |
| 70 | 83.3 | 71.8 | 11.5 | 60.4 | 41.9 | 55.6 | 63.0 | 123.0 | 51.2 |
| 71 | 78.8 | 71.8 | 7.0 | 54.6 | 43.8 | 40.9 | 74.9 | 114.0 | 65.7 |
| 72 | 79.4 | 66.6 | 12.8 | 55.7 | 30.8 | 49.3 | 68.4 | 122.4 | 55.9 |
| 73 | 78.6 | 68.7 | 9.9 | 57.2 | 35.6 | 60.3 | 68.9 | 128.9 | 53.5 |
| 74 | 86.7 | 77.0 | 9.6 | 67.8 | 53.2 | 39.3 | 72.6 | 116.6 | 62.3 |
| 75 | 82.3 | 72.0 | 10.3 | 60.6 | 37.2 | 57.6 | 64.0 | 123.5 | 51.8 |
