# Supplementary material for: Cephalometric Analysis of the Facial Skeletal Morphology of Female Patients Exhibiting Skeletal Class II Deformity with and without Temporomandibular Joint Osteoarthrosis
Source: PLoS One. 2015 Oct 16;10(10):e0139743. doi: 10.1371/journal.pone.0139743 (PMC4608765; doi:10.1371/journal.pone.0139743)
Supplement: S2 Table — (DOCX) [file pone.0139743.s002.docx]

**Supporting Information**

**(S2)**

**Table 1.Repeated cephalometric measurements at least 2 weeks apart to assess reliability of the method.**

|  | SNA 1 | SNA 2 | SNB1 | SNB2 | A1 | A2 | Pog 1 | Pog2 | MP - SN1 | MP - SN2 | SGo1 | SGo2 | N-Me1 | N-Me2 |
| --- | --- | --- | --- | --- | --- | --- | --- | --- | --- | --- | --- | --- | --- | --- |
| 1 | 85.2 | 84.5 | 73.7 | 72.6 | 65.2 | 61.8 | 51.9 | 48.9 | 47.8 | 49.0 | 84.5 | 84.1 | 137.9 | 137.9 |
| 2 | 77.7 | 77.8 | 69.7 | 69.4 | 58.8 | 59.7 | 43.6 | 43.7 | 42.9 | 44.9 | 68.7 | 66.0 | 116.2 | 117.6 |
| 3 | 85.3 | 84.8 | 77.1 | 76.7 | 67.4 | 67.3 | 56.1 | 56.7 | 36.0 | 35.5 | 78.8 | 78.5 | 122.9 | 122.2 |
| 4 | 84.3 | 83.5 | 74.8 | 74.0 | 64.0 | 63.7 | 46.4 | 45.7 | 42.8 | 43.4 | 84.7 | 84.0 | 135.3 | 135.5 |
| 5 | 76.4 | 75.8 | 68.0 | 68.4 | 59.9 | 59.0 | 39.7 | 40.4 | 41.0 | 40.5 | 85.5 | 84.6 | 133.6 | 132.2 |
| 6 | 78.8 | 78.5 | 71.6 | 71.3 | 55.8 | 54.9 | 40.7 | 40.0 | 47.8 | 47.5 | 69.1 | 69.1 | 121.4 | 120.6 |
| 7 | 84.3 | 83.9 | 74.8 | 74.4 | 61.4 | 61.1 | 45.8 | 45.6 | 42.8 | 43.3 | 84.7 | 85.7 | 135.1 | 135.4 |
| 8 | 80.4 | 79.6 | 70.1 | 69.6 | 61.2 | 60.5 | 43.8 | 41.9 | 41.5 | 41.7 | 68.3 | 69.1 | 118.7 | 119.8 |
| 9 | 81.9 | 82.6 | 70.3 | 70.7 | 61.8 | 62.5 | 42.8 | 43.1 | 38.8 | 38.5 | 78.3 | 77.3 | 120.6 | 120.2 |
| 10 | 81.0 | 80.8 | 71.2 | 70.7 | 66.4 | 66.6 | 41.8 | 40.6 | 39.1 | 38.7 | 68.3 | 68.9 | 129.7 | 130.1 |
| 11 | 81.5 | 80.7 | 72.1 | 71.9 | 59.0 | 58.4 | 42.0 | 42.3 | 45.4 | 45.0 | 70.1 | 69.8 | 120.9 | 120.6 |
| 12 | 81.7 | 83.0 | 74.1 | 74.5 | 59.6 | 60.3 | 42.0 | 41.2 | 49.1 | 48.3 | 72.4 | 73.4 | 128.8 | 128.9 |
| 13 | 74.3 | 74.8 | 67.2 | 67.3 | 51.1 | 51.4 | 33.3 | 33.3 | 51.0 | 51.8 | 61.2 | 59.6 | 118.9 | 118.1 |
| 14 | 86.0 | 85.3 | 72.0 | 71.9 | 67.8 | 68.0 | 44.0 | 43.1 | 47.3 | 48.5 | 71.3 | 72.4 | 124.9 | 126.8 |
| 15 | 80.1 | 80.4 | 71.8 | 72.6 | 61.0 | 61.9 | 46.0 | 47.1 | 45.5 | 43.9 | 70.0 | 70.8 | 121.0 | 120.8 |
